# DPF2 overexpression correlates with immune infiltration and dismal prognosis in hepatocellular carcinoma

**DOI:** 10.7150/jca.97437

**Published:** 2024-07-02

**Authors:** Kejian Yang, Jusen Nong, Haixiang Xie, Zuyin Wan, Xin Zhou, Junqi Liu, Chongjiu Qin, Jianzhu Luo, Guangzhi Zhu, Tao Peng

**Affiliations:** 1 Department of Hepatobiliary Surgery, The First Affiliated Hospital of Guangxi Medical University, Nanning, Guangxi Zhuang Autonomous Region, People's Republic of China.; 2 Guangxi Key Laboratory of Enhanced Recovery after Surgery for Gastrointestinal Cancer, Nanning, Guangxi Zhuang Autonomous Region, People's Republic of China.; 3 Key Laboratory of early Prevention & Treatment for regional High Frequency Tumor (Guangxi Medical University), Ministry of Education, Nanning, Guangxi Zhuang Autonomous Region, People's Republic of China.

**Keywords:** DPF2, hepatocellular carcinoma (HCC), tumor prognosis, immune infiltration

## Abstract

**Background:** Double plant homeodomain finger 2 (DPF2), belonging to the d4 family of structural domains, has been associated with various human malignancies. However, its impact on hepatocellular carcinoma (HCC) remains unclear. The objective of this study is to elucidate the role of DPF2 in the diagnosis and prognosis of HCC.

**Methods:** DPF2 gene expression in HCC and adjacent tissues was analyzed using Gene Expression Omnibus (GEO) and The Cancer Genome Atlas (TCGA) databases, validated by immunohistochemical staining of Guangxi specimens and data from the Human Protein Atlas (HPA). Gene Ontology (GO), Kyoto Encyclopedia of Genes and Genome (KEGG), and Gene Set Enrichment Analysis (GSEA) were used to identify DPF2's potential pathways and functions in HCC. DPF2's mutation and methylation statuses were assessed via cBioPortal and MethSurv. The association between DPF2 and immune infiltration was investigated by TIMER. The prognostic value of DPF2 in HCC was established through Kaplan-Meier and Cox regression analyses.

**Results:** DPF2 levels were significantly higher in HCC than normal tissues (p<0.001), correlating with more severe HCC features (p<0.05). Higher DPF2 expression predicted poorer overall survival (OS), disease-specific survival (DSS), and progression-free interval (PFI). DPF2 involvement was noted in critical signaling pathways including the cell cycle and Wnt. It also correlated with T helper cells, Th2 cells, and immune checkpoints like CTLA-4, PD-1, and PD-L1.

**Conclusion:** High DPF2 expression, associated with poor HCC prognosis, may disrupt tumor immune balance and promote immune evasion. DPF2 could potentially be utilized as a biomarker for diagnosing and prognosticating hepatocellular carcinoma.

## 1. Introduction

Hepatocellular carcinoma (HCC), a notably aggressive and lethal malignancy, represents a significant global health burden. According to the World Health Organization (WHO), liver cancer is ranked sixth in global cancer incidence (4.7%) and third in mortality (8.3%). Among males, it is fifth in incidence (6.3%) and second in mortality (10.5%). In 2020, it is estimated that approximately 900,000 individuals were diagnosed with liver cancer diagnosis, and 830,000 succumbed to the disease 1, 2. Notably, there are significant geographical disparities in the morbidity and mortality rates of HCC, particularly in Asia and China, where liver cancer ranks fifth in incidence and second in mortality 3-5. Most HCC diagnoses occur at advanced stages, contributing to the high mortality rate associated with this malignancy. Current early detection strategies for liver cancer, such as tumor marker analysis and imaging examinations, are traditional and require improvements in accuracy and sensitivity 6, 7. Consequently, there is an urgent need for innovative and efficient diagnostic techniques to enhance early detection rates and the precision of prognostic evaluations for HCC.

The DPF (double PHD finger) domain, a member of the PHD (plant homeodomain) finger group, is predominantly found in proteins that interact with the N-terminal fragment of histones. This domain is crucial for the transcriptional activation of a wide array of genes, thereby influencing mammalian tissue development and differentiation. Mutations or aberrant expression of the DPF domain have been linked to the development of various types of cancers 8. Proteins encoded by the DPF2 gene belong to the d4 structural domain family, characterized by a zinc-finger-like structural motif. These proteins have been shown to efficiently bind to the SWI/SNF complex, thereby activating non-classical NF-κB transcription and its associated oncogenic activity 9. Additionally, genes from the DPF family have been associated with various cancers, including acute myeloid leukemia (AML), breast cancer, cervical cancer, and renal cell carcinoma 10-14.

Research has shown that reducing DPF2 impairs cell viability and induces apoptosis in human pancreatic cancer cells, suggesting a crucial role for DPF2 in the initiation and progression of these cells 15. However, studies on DPF2's role in hepatocellular carcinoma are limited, making its potential impact on the development and progression of this disease uncertain.

The objective of this research is to examine the expression and prognostic implications of DPF2 in hepatocellular carcinoma through comprehensive bioinformatics analysis and immunohistochemical experimental validation. It involves exploring the biological function of DPF2 and the signaling pathways involved in the pathogenesis and progression of HCC. Additionally, the study assesses the gene mutation and DNA methylation status of DPF2 and examines its role in modulating the tumor immune microenvironment. The ultimate goal is to provide novel insights and methodologies for the diagnosis and therapeutic intervention of HCC.

## 2. Materials and Methods

### 2.1 Data processing

Transcriptomic and clinical data for HCC and adjacent normal liver tissue were obtained from the TCGA database (https://portal.gdc.cancer.gov/) (Table 1), with RNA-seq data normalized to TPM format. Additional TPM-standardized RNA-seq data were sourced from the Genotype-Tissue Expression (GTEx) database for a comprehensive pan-cancer analysis. mRNA expression data from the GSE14520_3921 (Table S1), GSE14520_571 (Table S2), GSE76427, and GSE121248 datasets of the GEO database were downloaded from the NCBI database (https://www.ncbi.nlm.nih.gov/) for external validation of DPF2 expression differences and survival analysis.

### 2.2 DPF2 protein expression and HPA

DPF2 protein expression variations between HCC cells and normal hepatocytes were assessed using data from the Clinical Proteomic Tumor Analysis Consortium (CPTAC) available on the UALCAN website (https://ualcan.path.uab.edu/analysis-prot.html). DPF2 immunohistochemical data in HCC specimens were obtained from the Human Protein Atlas (HPA) database. 16.

### 2.3 Survival analysis

Survival analysis was performed on the TCGA_LIHC, GSE14520_3921, and GSE14520_571 databases using Kaplan-Meier and Cox regression models. The databases were stratified into groups with high and low DPF2 expression levels using the 'surv_cutpoint' algorithm from the survminer package in R 17. The impact of clinical variables on outcomes was evaluated through univariate and multivariate Cox analyses. Prognostic variables with a p-value less than 0.1 from the univariate analysis were included in the multivariate analysis. The results of the regression were visualized as forest plots using ggplot2.

### 2.4 Building and validating nomogram

Based on the independent prognostic factors derived from the COX analysis, we developed nomograms that predict survival probabilities. These nomograms use scaled line segments to integrate multiple predictors and are plotted on the same plane at a specific scale, effectively illustrating the interrelationships between the predictor variables in the predictive model. The validity of the nomograms was assessed by creating calibration plots using the RMS software package to analyze how well the model fits the actual situation.

### 2.5 Differentially Expressed Gene analysis

Based on the median DPF2 expression in TCGA_LIHC, samples were categorized into groups with high and low DPF2 expression. The 'DESeq2' package in R was used to conduct differential gene expression (DEG) analysis 17, with an adjusted p-value < 0.05 and |log2FoldChange| > 2 as the thresholds for identifying DEGs. The association between DPF2 expression and the top 5 upregulated and downregulated DEGs was investigated using Spearman correlation analysis. DEGs were visualized as volcano plots.

### 2.6 Functional Enrichment Analysis

GO, KEGG, and GSEA analyses were conducted on DEGs using the 'clusterProfiler' package. For GSEA analysis, the gene sets c2.cp.all.v2022.1.Hs.symbols.gmt and c5.all.v2022.1.Hs.symbols.gmt were used 18. Significance was established with an adjusted p-value<0.05 and False Discovery Rate (FDR) <0.25. Results were visualized using ggplot2.

### 2.7 Protein-protein interaction network analysis

Protein interactions were scrutinized using STRING with a confidence score exceeding 0.7, resulting in a PPI network comprising DPF2 proteins and 15 associated proteins 19. Additionally, the interactions and functions of the DPF2 gene were analyzed using GeneMANIA (http://genemania.org) 20.

### 2.8 Gene mutations and DNA methylation

DPF2 mutations and copy number variations (CNV) were explored via cBioPortal, examining the correlation between DPF2 genetic alterations and HCC prognosis 21. DPF2 promoter methylation was investigated using UALCAN 22, while MethSurv assessed the prognostic significance of DPF2 methylation levels 23.

### 2.9 Immune infiltration analysis

Enrichment scores for 24 types of immune cells were determined using the ssGSEA algorithm through GSVA. The correlation between DPF2 expression and immune cell infiltration, as well as its relationship with TP53 and immune checkpoints—CTLA4, PDCD1/PD1, CD274/PDL1, was assessed using Spearman analysis 24, 25. Differences in immune cell infiltration between groups with high and low DPF2 expression were analyzed using the Wilcoxon rank-sum test.

### 2.10 Pathologic sample collection

Seventeen pairs of HCC and corresponding paracarcinoma tissues were collected from HCC patients at the First Hospital of Guangxi Medical University. Inclusion criteria included: 1. Patients with primary hepatocellular carcinoma treated for the first time; 2. Patients treated with partial hepatectomy; 3. Patients who have not undergone interventional therapy, targeted therapy, or immunotherapy before surgery. Exclusion criteria: involved patients with a history of other tumors besides hepatocellular carcinoma. The study, approved by the hospital's Ethics Committee (NO. 2024-E068-01) in accordance with the Declaration of Helsinki, secured written informed consent from all participants.

### 2.11 Immunohistochemistry

Paired tumors and para-cancerous tissues were processed according to the following protocols: 1. Tissue Preparation: Sections were deparaffinized in xylene and hydrated through a graded series of ethanol (100%, 95%, 85%, 75%). 2. Antigen Repair: Antigen repair was performed using a citrate buffer (pH 6.0) in a microwave (95°C) for 15 minutes. 3. Blocking: After cooling, sections were treated with an appropriate amount of endogenous peroxidase blocker (PV-9000, ZSGB-Bio, China), and incubated at room temperature for 10 minutes. 4. Antibody Incubation: Sections were incubated with a primary antibody against DPF2 (Proteintech, No. 12111-1-AP, 1:150) overnight at 4°C. Sections were then incubated for 20 minutes at room temperature with appropriate amounts of reaction enhancement solution and enhancer-labeled sheep anti-mouse/rabbit IgG polymers (PV-9000, ZSGB-Bio, China). 5. Visualization: Visualization was achieved using DAB (diaminobenzidine) as the chromogen. 6. Counterstaining: Sections were counterstained with hematoxylin to highlight nuclei. 7. Mounting: Sections were dehydrated, cleared, and mounted for microscopic evaluation. Staining results were analyzed using Image-J software. The Average Optical Density (AOD) of DPF2-positive stains was calculated for semi-quantitative analysis: AOD=Integrated Optical Density/Area Analyzed 26-28.

### 2.12 Statistical analysis

IBM SPSS Statistics 26 and R (version 4.2.1) were employed for data analysis. The Wilcoxon rank-sum test was used to assess the significance of DPF2 expression in unpaired tissues, while the paired samples t-test was used for paired tissues. The association between clinical characteristics and DPF2 expression was examined using the Wilcoxon rank-sum test and logistic regression. Spearman's rank correlation was utilized to analyze the relationship between the two groups. Statistical significance was determined by p-value<0.05.

## 3. Results

### 3.1 High expression of DPF2 in hepatocellular carcinoma

**Figure 1:**
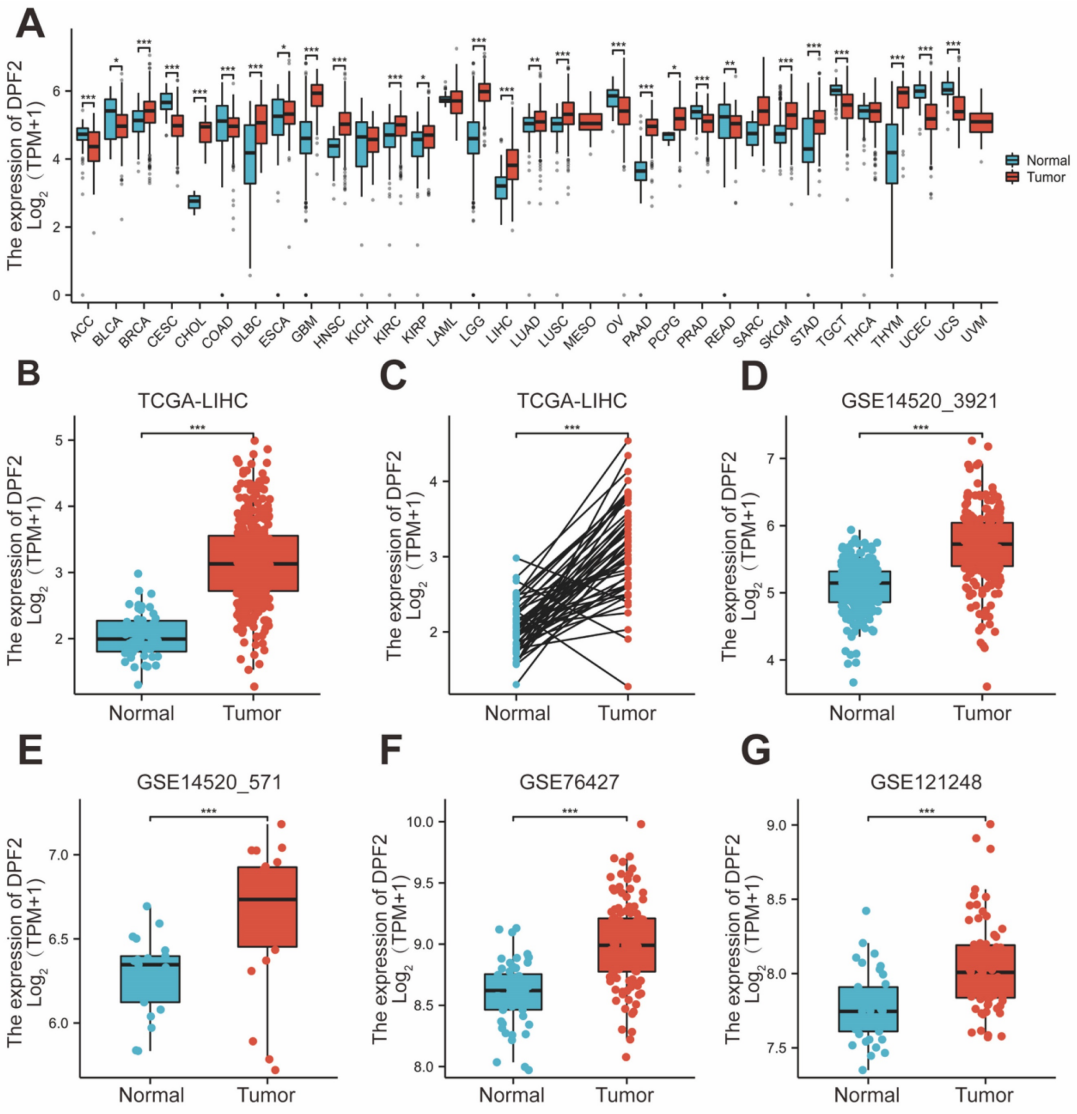
** Expression of DPF2 in different types of tumors and liver cancer.** (A) Pan-cancer analysis of DPF2 in TCGA and GTEx databases. (B) TCGA database of HCC and unpaired normal liver tissues. (C) TCGA database of HCC and paired normal liver tissues. (D) GSE14520_3921. (E) GSE14520_571. (F) GSE76427. (G) GSE121248. TCGA, The Cancer Genome Atlas; GTEx, Genotype Tissue Expression Project. ns: p ≥ 0.05; *p < 0.05; **p < 0.01; ***p < 0.001.

Pan-cancer analysis, leveraging the TCGA and GTEx databases, revealed that DPF2 was markedly overexpressed in a majority of cancer types, including hepatocellular carcinoma, pancreatic cancer, breast cancer, cholangiocarcinoma, melanoma, and squamous cell carcinoma of the head and neck (Figure 1A). DPF2 expression was notably higher in patients with hepatocellular carcinoma compared to normal hepatocyte tissues (p<0.001) (Figure 1B). In a comparison of 50 pairs of hepatocellular carcinoma and corresponding paracarcinomatous tissues, significant overexpression of DPF2 was observed in the hepatocellular carcinoma tissues (p<0.001) (Figure 1C). This heightened expression of DPF2 in hepatocellular carcinoma tissues (p<0.001) was corroborated at the transcriptome level in the GSE14520_3921, GSE14520_571, GSE76427, and GSE121248 datasets (Figure 1D-G).

**Figure 2:**
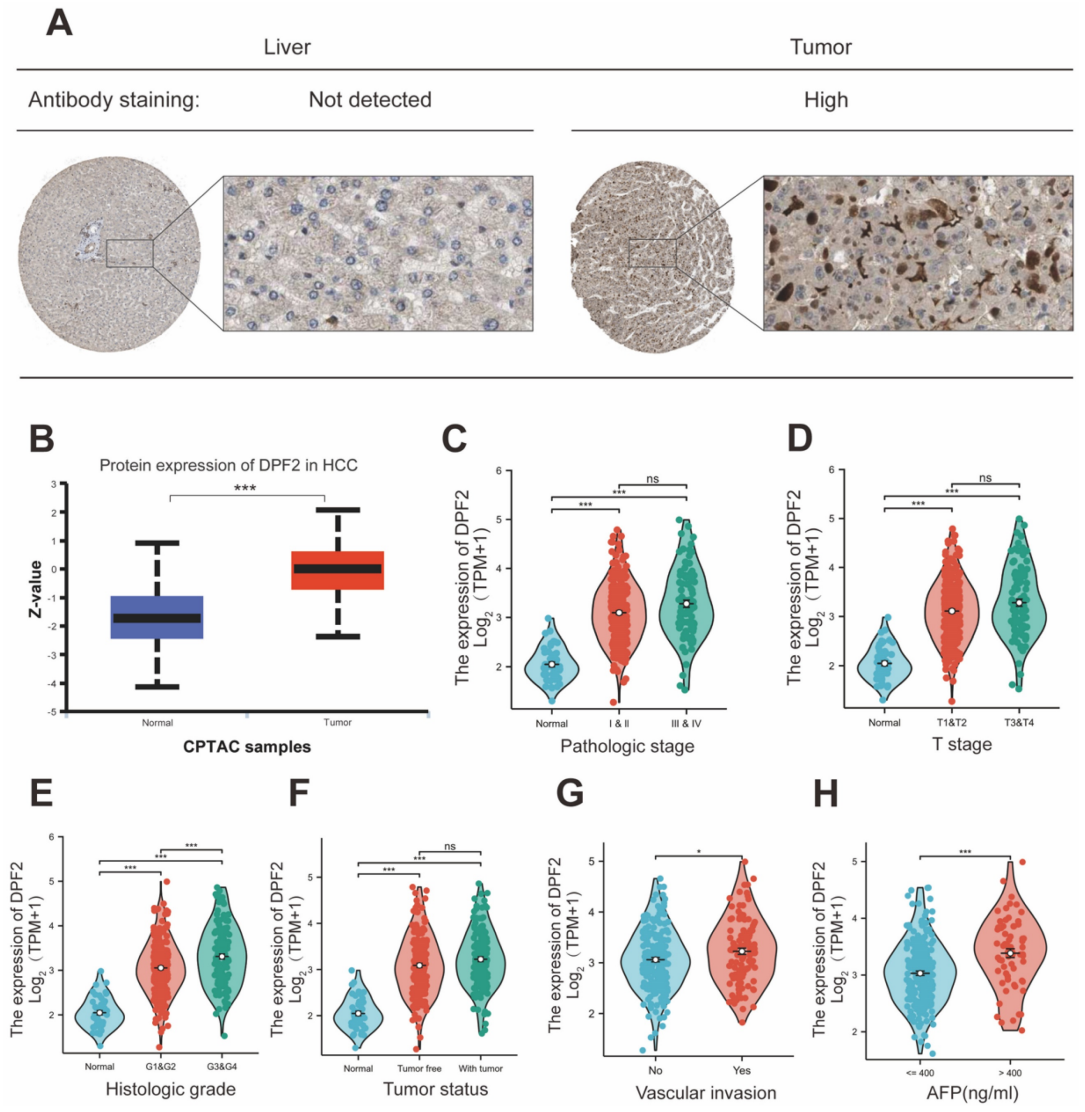
** Expression of DPF2 at the protein level and relationship between DPF2 and clinicopathological features.** (A) Typical immunohistochemical images of DPF2 expression in HCC tissues and normal liver tissues from the HPA database. (B) DPF2 protein expression in HCC tissues and normal liver tissues from the CPTAC database in the UALCAN website. (C) Pathological stage. (D) T stage. (E) Histologic grade. (F) Tumor status. (G) Vascular invasion. (H) AFP. AFP, alpha-fetoprotein. ns: p ≥ 0.05; *p < 0.05; **p < 0.01; ***p < 0.001.

Further characterization of DPF2 expression at the protein level was conducted through Immunohistochemistry (IHC) of hepatocellular carcinoma and normal liver tissues, sourced from the HPA website. This analysis demonstrated that the IHC staining intensity of DPF2 in hepatocellular carcinoma was significantly greater than that in paracancerous tissues (Figure 2A). Additionally, results from the CPTAC database showed a significant increase in the protein expression level of DPF2 in hepatocellular carcinoma tissues compared to normal hepatocyte tissues (Figure 2B).

### 3.2 High DPF2 expression is associated with adverse clinicopathologic features

Elevated expression of DPF2 was significantly correlated with a more advanced stage of HCC, residual tumor status, vascular invasion, and higher AFP levels (all p<0.05) (Table 1, Figure 2C-H). Additionally, the outcomes of the univariate logistic regression analysis showed a strong correlation between DPF2 expression and clinical pathology characteristics, particularly AFP (OR=2.952, 95% CI=1.633-5.334, p<0.001), and histological stage (OR=2.011, 95% CI=1.307-3.095, p=0.001). Furthermore, DPF2 may be associated with age (OR=0.686, 95% CI=0.456-1.032, p=0.070), race (OR=1.461, 95% CI=0.966-2.211, p=0.073), tumor status (OR=1.495, 95% CI=0.980-2.280, p=0.062), and vascular invasion (OR=1.570, 95% CI=0.986-2.499, p=0.057), although these correlations were not statistically significant (Table 2).

### 3.3 High DPF2 expression is associated with poorer prognosis in HCC

**Figure 3:**
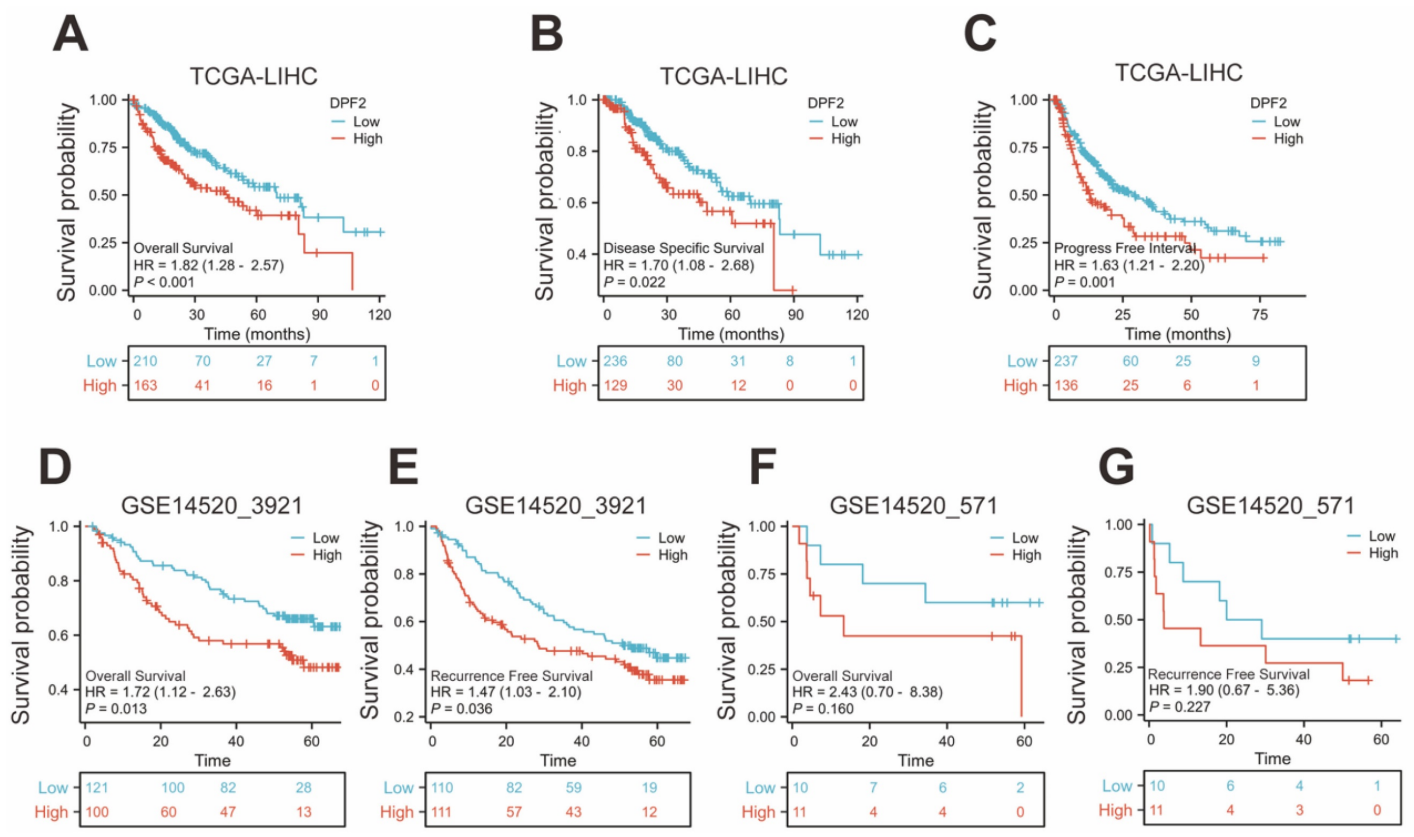
**High expression of DPF2 was associated with poor prognosis.** Kaplan-Meier survival curves of DPF2 in (A) OS, (B) DSS and (C) PFI in The Cancer Genome Atlas (TCGA), (D) OS and (E) RFS in GSE14520_3921, (F) OS and (G) RFS in GSE14520_571 subgroups. OS, Overall Survival. DSS, Disease Free Survival. PFI: Progression Free Interval. RFS, Recurrence Free Survival.

The Kaplan-Meier method was used to evaluate the prognostic relationship between DPF2 expression and hepatocellular carcinoma. The 'surv_cutpoint' algorithm was used to divide patients into groups with high and low DPF2 expression 29. The findings indicated that high DPF2 expression correlated with poorer overall survival (OS) (HR=1.82, 95% CI=1.28-2.57, p<0.001), disease-specific survival (DSS) (HR=1.70, 95% CI=1.08-2.68, p=0.022), and progression-free interval (PFI) (HR=1.63, 95% CI=1.21-2.20, p=0.001) (Figure 3A-C). Further Kaplan-Meier analysis of the GSE14520_3921 and GSE14520_571 datasets demonstrated that high DPF2 expression in GSE14520_3921 was associated with poorer OS (HR=1.72, 95% CI=1.12-2.63, p=0.013) and recurrence-free survival (RFS) (HR=1.47, 95% CI=1.03-2.10, p=0.036) (Figure 3D-E). In the GSE14520_571 dataset, similar results were observed, but the differences in OS (p=0.160) and RFS (p=0.227) were not statistically significant, likely due to the small sample size (Figure 3F-G).

Subsequent analysis of overall survival differences associated with DPF2 expression in various clinical subgroups revealed that patients with high DPF2 expression had a poorer prognosis across multiple categories: AFP ≤ 400, T1 and T2, T3 and T4, G1 and G2, G3 and G4, and stage I and II, as well as stage III and IV subgroups (all p<0.05) (Figure S1).

### 3.4 Diagnostic and prognostic value of DPF2 in HCC

**Figure 4:**
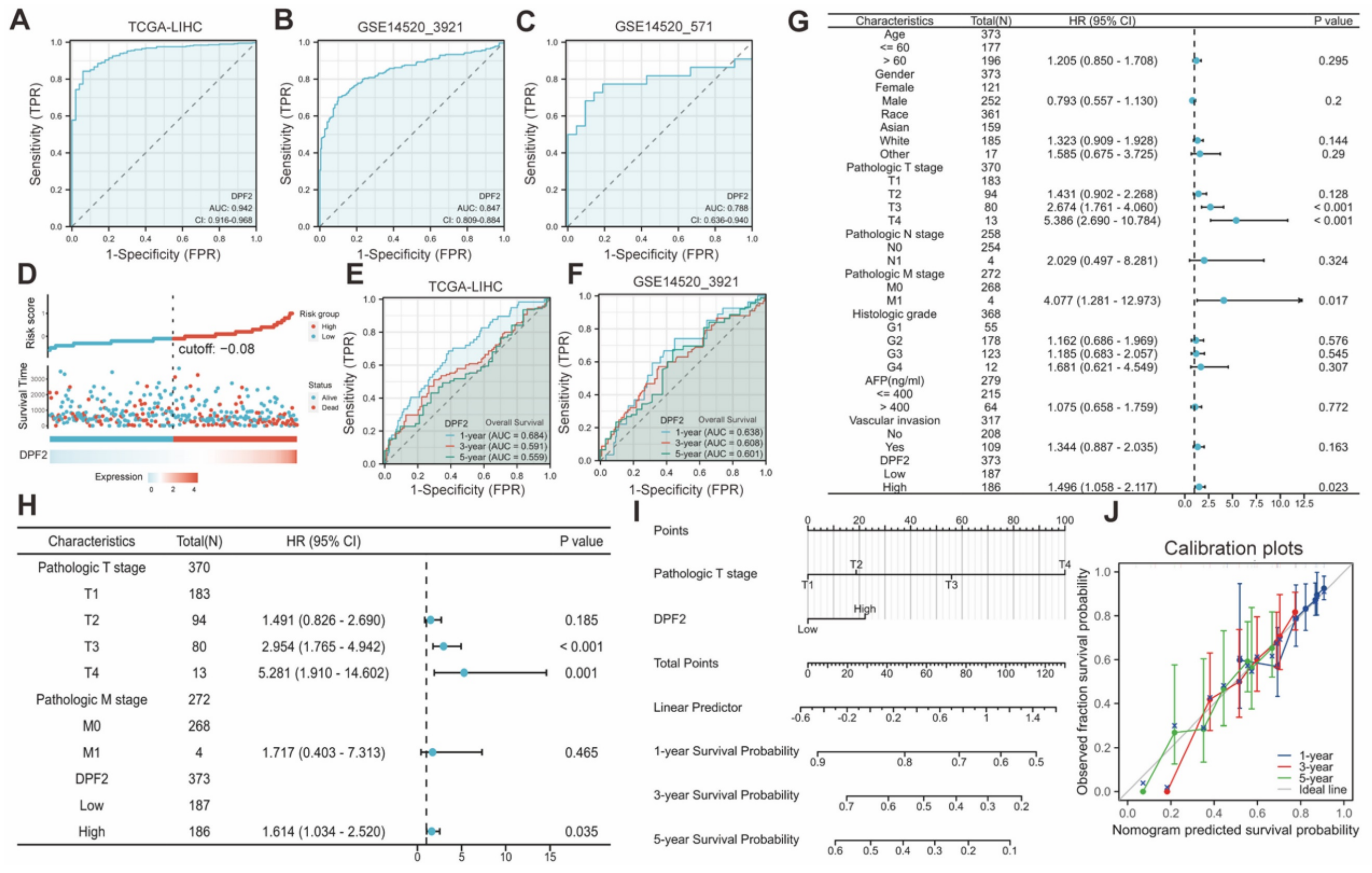
** Predictive ability of DPF2 for hepatocellular carcinoma (HCC).** Diagnostic ROC curves in (A) The Cancer Genome Atlas (TCGA), (B) GSE14520_3921, (C) GSE14520_571. (D) Risk score, survival time distribution, and gene expression heat map of DPF2 in TCGA. Predictive power of DPF2 for 1-, 3-, and 5-year overall survival (OS) by time-dependent ROC analysis in (E) TCGA, and (F) GSE14520_3921. (G) Forest map based on Univariate Cox analysis for overall survival. (H) Forest map based on Multivariate Cox analysis for overall survival. (I) Prediction of 1-, 3-, and 5-year OS by nomogram. (J) Calibration plots were used to validate the nomogram model.

DPF2's diagnostic potential in HCC was evaluated by performing ROC analysis on the TCGA, GSE14520_3921, and GSE14520_571 databases. The findings indicated that DPF2 demonstrated superior prognostic efficacy across all datasets, with AUC values of 0.942 for TCGA (Figure 4A), 0.847 for GSE14520_3921 (Figure 4B), and 0.788 for GSE14520_571 (Figure 4C). Additional analyses of other datasets and clinicopathological subgroups confirmed DPF2's robust predictive capacity (Figure S2). The predictive value of DPF2 expression in the prognosis of HCC was further assessed by building a risk score model. The data showed that patients in the high-risk score cohort experienced endpoint events within a shorter timeframe, highlighting the link between increased DPF2 expression and poorer HCC prognosis, and affirming its prognostic relevance in HCC (Figure 4D). Time-dependent ROC curves were generated from the TCGA and GSE14520_3921 datasets further supported the prognostic value of DPF2 in predicting 1-, 3-, and 5-year survival rates (Figure 4E-F).

Univariate and multivariate Cox regression analyses were conducted to identify the prognostic determinants of HCC in relation to DPF2 expression. Univariate analysis revealed a significant correlation between DPF2 overexpression (high vs. low, HR 1.496, p = 0.023), T stage (T3 vs. T1, HR 2.674, p < 0.001; T4 vs. T1, HR 5.386, p < 0.001), and M stage (M1 vs. M0, HR 4.077, p = 0.017) with poorer OS in HCC patients (Figure 4G). Multivariate analysis further identified DPF2 overexpression (high vs. low, HR 1.614, p = 0.035) and T stage (T3 vs. T1, HR 2.954, p < 0.001; T4 vs. T1, HR 5.281, p = 0.001) as independent risk factors for OS in patients with HCC (Figure 4H).

We developed a nomogram based on independent risk factors for OS in HCC, such as T staging and DPF2 expression, to further confirm DPF2's prognostic impact on 1-, 3- and 5-year survival rates. The cumulative score, derived by aggregating the scores of each prognostic determinant, was used to forecast OS for HCC patients. The nomogram indicated that a higher total score corresponded to a less favorable prognosis (Figure 4I). Additionally, calibration curves were used to assess the discrepancy between the predicted probabilities by the nomogram at various time points and the actual probabilities. The results suggested that DPF2 expression could potentially offer a more accurate forecast of survival probabilities at 3 and 5 years compared to the prediction for the 1-year survival rate (Figure 4J).

### 3.5 DEG identification and functional enrichment analysis in HCC patients

**Figure 5:**
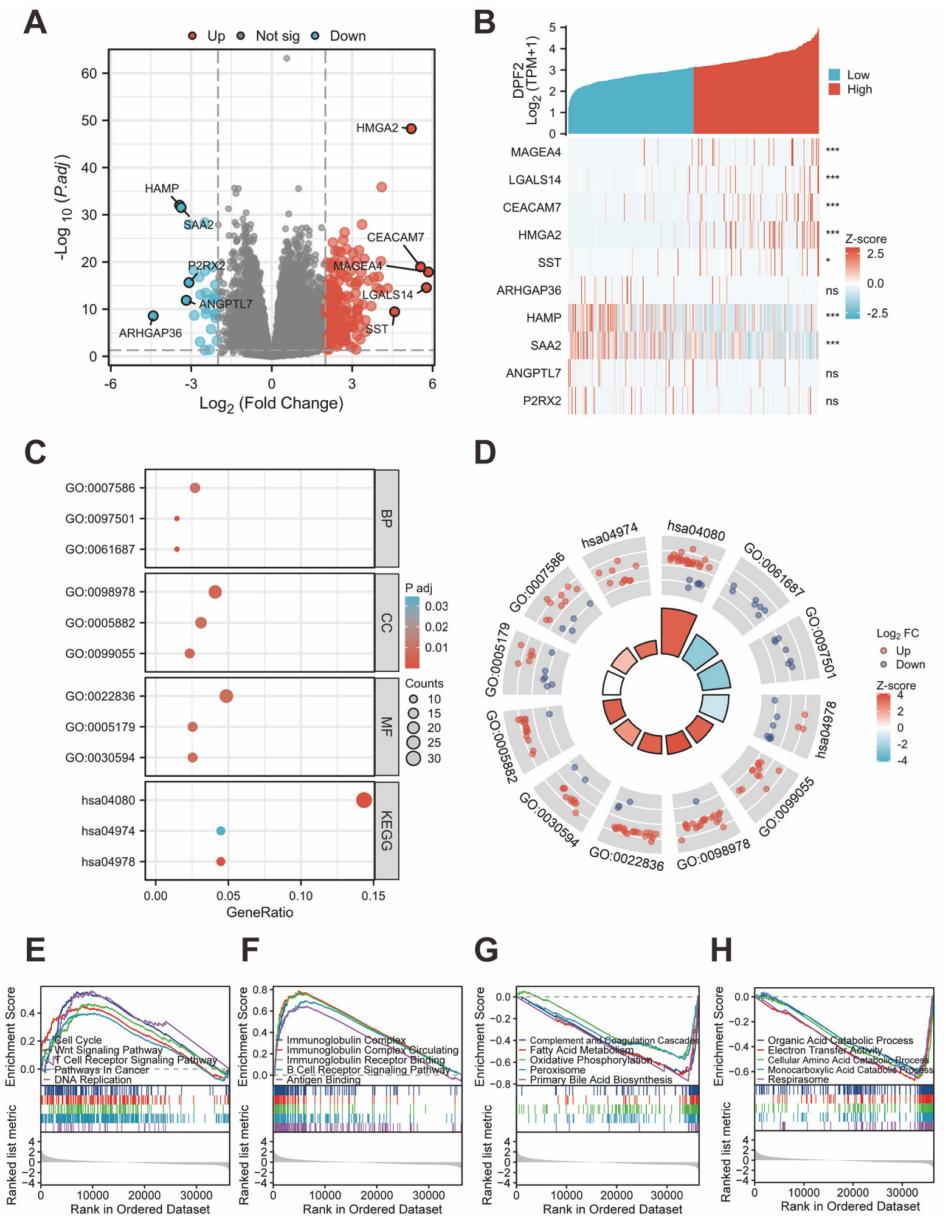
** Analysis of differentially expressed genes and functional enrichment of DPF2 in HCC.** (A) Volcano plot showing the DEGs between DPF2 high and DPF2 low groups. (B) Heat map showing the top five upregulated and downregulated genes with DPF2 expression. (C) Bubble plot of GO and KEGG enrichment analysis. (D) Circle diagram showing the GO and KEGG terms corresponding to the DEGs. (E-F) Enrichment results of GSEA gene set in DPF2 high expression group. (G-H) Enrichment results of GSEA gene set in the DPF2 low expression group. GO:Gene Ontology; KEGG: Kyoto Encyclopedia of Genes and Genomes. ns: p ≥ 0.05; *p < 0.05; **p < 0.01; ***p < 0.001.

Between the cohorts with high and low DPF2 expression, a total of 245 transcriptomic genes showed differential expression, including 216 genes that were upregulated and 29 genes that were downregulated (with an adjusted p-value<0.05 and |log_2_Foldchange |>2) (Figure 5A). The top five upregulated and downregulated differential genes (MAGEA4, LGALS14, CEACAM7, HMGA2, SST, ARHGAP36, HAMP, SAA2, ANGPTL7, P2RX2) were subjected to further analysis based on their adjusted p-values. Heatmaps were employed to delineate the relationship between these genes and DPF2 expression (Figure 5B). Functional annotation of the differentially expressed genes (DEGs) was carried out using GO and KEGG enrichment analyses. The results indicated that the primary biological processes (BP) involved digestion, metal ion stress response, and inorganic compound detoxification. The main cellular components (CC) were associated with glutamatergic synapse, intermediate filament, and integral components of the postsynaptic membrane. The key molecular functions (MF) involved gated channel activity, hormone activity, and neurotransmitter receptor activity. KEGG analysis revealed that these differential genes were predominantly implicated in pathways such as Neuroactive ligand-receptor interaction, Protein digestion and absorption, and mineral absorption (Figure 5C-D).

We investigated the association between DPF2 expression and diverse biological processes by conducting GSEA using both c2.cp.all.v2022.1.Hs.symbols.gmt and c5.all.v2022.1.Hs.symbols.gmt datasets. The findings revealed that elevated DPF2 expression was significantly correlated with processes such as the cell cycle, Wnt signaling pathway, T cell receptor signaling pathway, cancer pathways, DNA replication, immunoglobulin complex, circulating immunoglobulin complex, immunoglobulin receptor binding, B cell receptor signaling pathway, and antigen-binding (Figure 5E-F). Conversely, a significant association was observed between reduced DPF2 expression and various processes and pathways, including the complement and coagulation cascades, fatty acid metabolism, oxidative phosphorylation, peroxisome, primary bile acid biosynthesis, organic acid catabolic process, electron transfer activity, cellular amino acid catabolic process, monocarboxylic acid catabolic process, and respirasome (Figure 5G-H).

### 3.6 PPI Network and Ferroptosis related analysis

**Figure 6:**
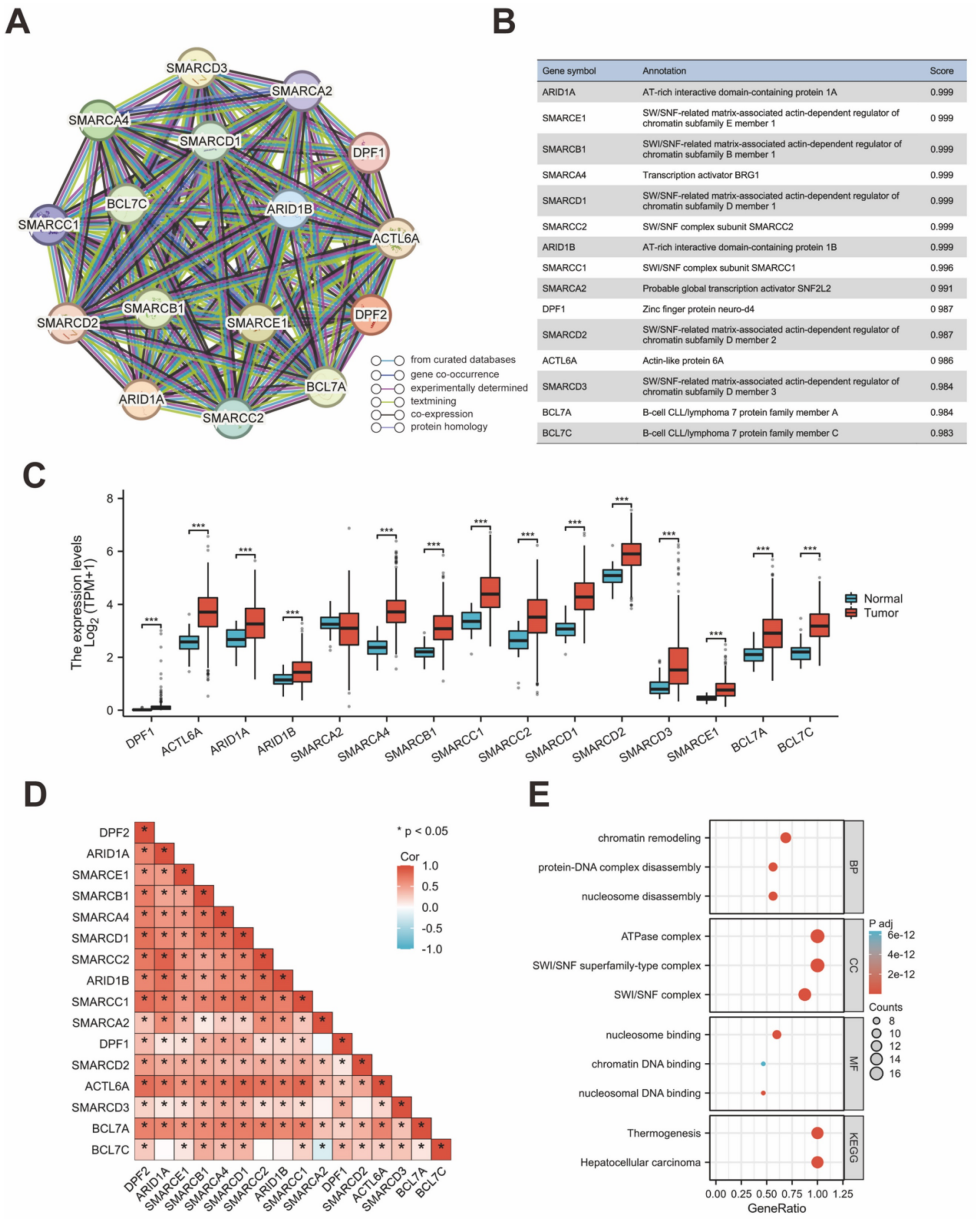
**DPF2-related genes and their functional analysis.** (A) PPI network of DPF2-related genes. (B) Annotation and correlation coefficients of 15 DPF2-related genes. (C) Expression of DPF2-related genes in HCC. (D) Correlation between DPF2 and related genes. (E) GO/KEGG functional enrichment analysis of DPF2-related genes. ns: p ≥ 0.05; *p < 0.05; **p < 0.01; ***p < 0.001.

**Figure 7:**
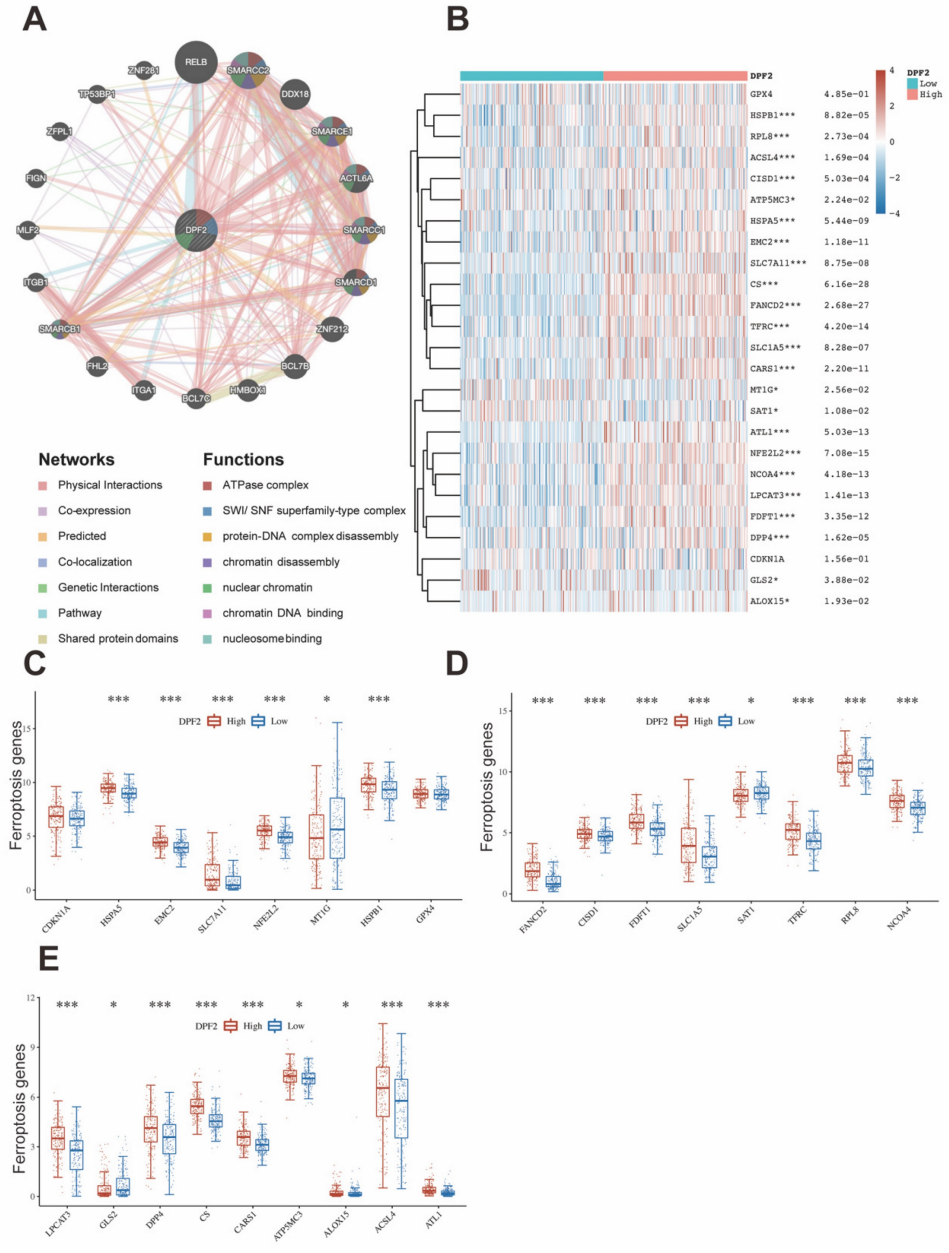
**Correlation of DPF2 with related genes in ferroptosis pathway.** (A) GeneMANIA Gene Interaction Network related to DPF2. (B) Heat map of the correlation between DPF2 expression and ferroptosis-related genes. (C-E) Expression of ferroptosis-related genes in the high and low DPF2 expression groups. ns: p ≥ 0.05; *p < 0.05; **p < 0.01; ***p < 0.001.

The protein interaction network for genes associated with DPF2 was constructed using the STRING database. This analysis highlighted ARID1A, SMARCE1, SMARCB1, SMARCA4, SMARCD1, SMARCC2, ARID1B, SMARCC1, SMARCA2, DPF1, SMARCD2, ACTL6A, SMARCD3, BCL7A, and BCL7C as the genes most significantly associated with DPF2 (Figure 6A-B,D). Comparative expression analysis of these genes in HCC showed that all related genes, except for SMARCA2, were significantly overexpressed in HCC compared to adjacent non-tumor tissues (p < 0.001) (Figure 6C). Subsequent prognostic analysis in HCC indicated that elevated expression of the majority of these genes was associated with poorer prognosis (Figure S3). Further analyses using GO and KEGG pathways revealed that DPF2 and its associated genes primarily participate in biological functions and pathways such as nucleosome disassembly, protein-DNA complex disassembly, chromatin remodeling, SWI/SNF complex, SWI/SNF superfamily-type complex, ATPase complex, nucleosome binding, nucleosomal DNA binding, chromatin DNA binding, Hepatocellular carcinoma, and Thermogenesis (Figure 6E). Additionally, the GeneMANIA database, which facilitates the discovery of functionally similar genes and prediction of gene function using extensive genomics and proteomics data 30, was used to validate the functions of DPF2-related genes, confirming their involvement in the aforementioned biological functions (Figure 7A).

Ferroptosis is an iron-dependent form of programmed cell death, distinct from other forms such as necrosis, apoptosis, and autophagy 31. Cancer cells, unlike normal cells, tend to utilize elevated levels of iron to promote growth and invasion 32. Ferroptosis has been shown to play a pivotal role in treating HCC and has emerged as a potent therapeutic approach 33, 34. Studies have established that certain genes are associated with ferroptosis in cancer 35. Using the TCGA database, our study examined the relationship between DPF2 and 25 genes related to ferroptosis. The analysis revealed a strong positive association between the expression of DPF2 and the genes HSPA5, EMC2, SLC7A11, NFE2L2, HSPB1, FANCD2, CISD1, FDFT1, SLC1A5, TFRC, RPL8, NCOA4, LPCAT3, DPP4, CS, CARS1, ATP5MC3, ALOX15, ACSL4, and ATL1. On the other hand, the expression of DPF2 was found to be negatively linked to MT1G, SAT1, and GLS2 (Figure 7B-E).

### 3.7 DPF2 gene mutation and methylation

**Figure 8:**
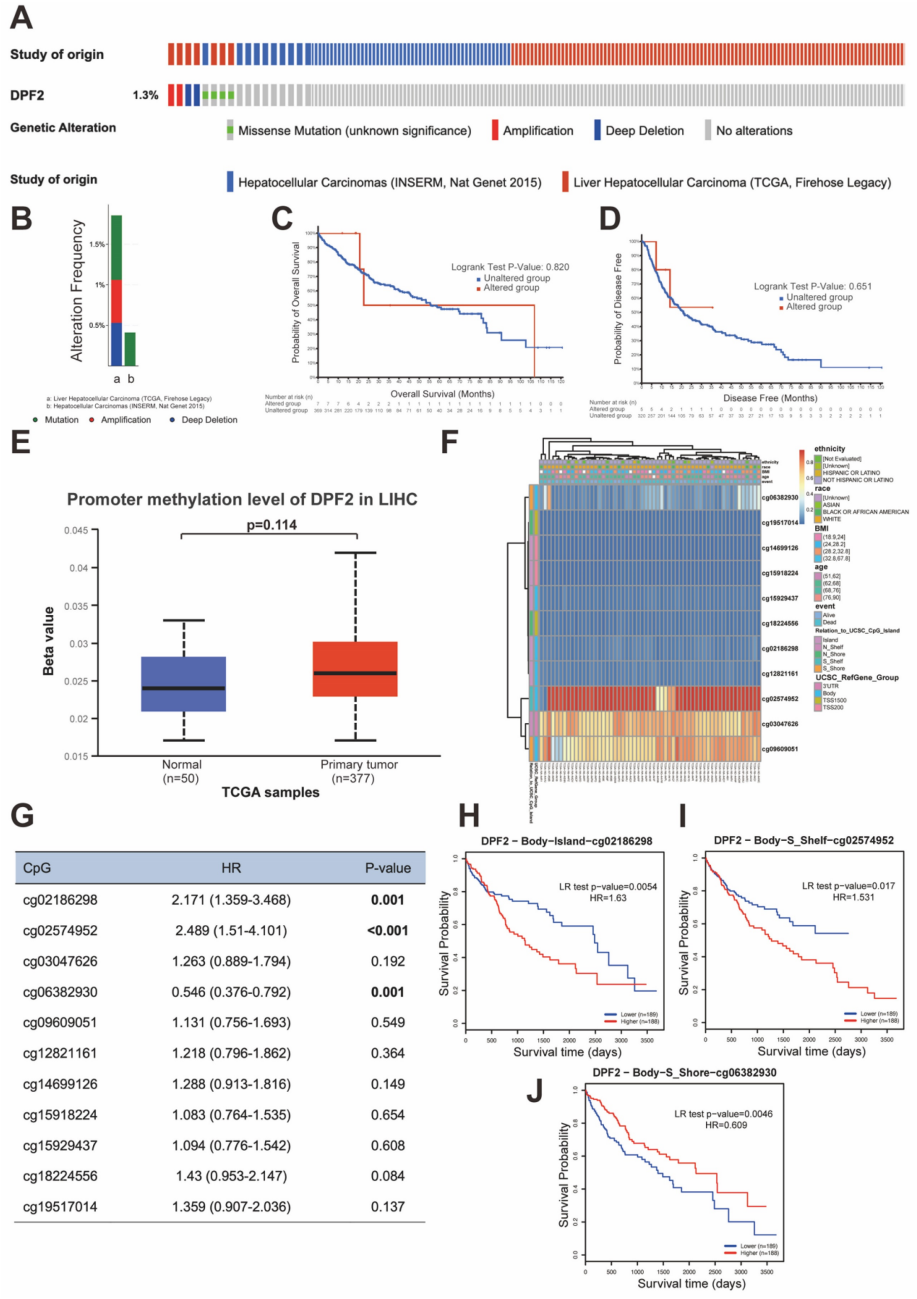
**Mutations and DNA methylation levels of DPF2 and their impact on the prognosis of HCC.** (A-B) Mutation levels of the DPF2 in cBioPortal OncoPrint.(C) Association between DPF2 gene mutation and overall survival (OS) in HCC. (D) Association between DPF2 gene mutation and disease-free survival (DFS) in HCC. (E) DPF2 methylation levels in HCC form the UALCAN database. (F) Correlation between DPF2 mRNA expression level and methylation level form the MethSurv database. (G) Correlation between DPF2 methylation level and prognosis of HCC. Kaplan-Meier survival curve of DPF2 in (H) cg02186298, (I) cg02574952, (J) cg06382930.

Genetic mutations are one of the primary etiological factors for cancer 36. In our study, we analyzed DPF2 mutations and copy number variations (CNVs) in two HCC datasets (INSERM, Nat Genet 2015, n=243; TCGA, Firehose Legacy, n=379) accessed from the cBioPortal. The study showed that the DPF2 gene exhibited missense mutation, amplification, and deep deletion at a frequency of 1.3% (Figure 8A-B). Further analyses using the Kaplan-Meier method indicated that mutations in the DPF2 gene did not correlate with differences in OS (p = 0.820) or DFS (p = 0.651) (Figure 8C-D). Additionally, it is well-established by various studies that abnormal DNA methylation significantly contributes to the early stages of HCC development 37, 38. We observed elevated DNA methylation levels of DPF2 at the promoter region in HCC tissues compared to normal liver tissues using the UALCAN database; however, this difference was not statistically significant (p = 0.114) (Figure 8E). A comprehensive examination of the DNA methylation level of the DPF2 gene and the prognostic relevance of CpG islands within the gene was conducted using the MethSurv database. The results indicated that the majority of these CpG sites exhibited hypomethylation (Figure 8F). Specifically, three CpG sites—cg02186298, cg02574952, and cg06382930—showed methylation levels correlated with the prognosis of HCC (Figure 8G). Hypermethylation of cg02186298 and cg02574952 was associated with a poorer prognosis, whereas hypomethylation of cg06382930 was also correlated with a poorer prognosis (Figure 8H-J).

### 3.8 DPF2 expression is considerably associated with immune infiltration and immune checkpoints

**Figure 9:**
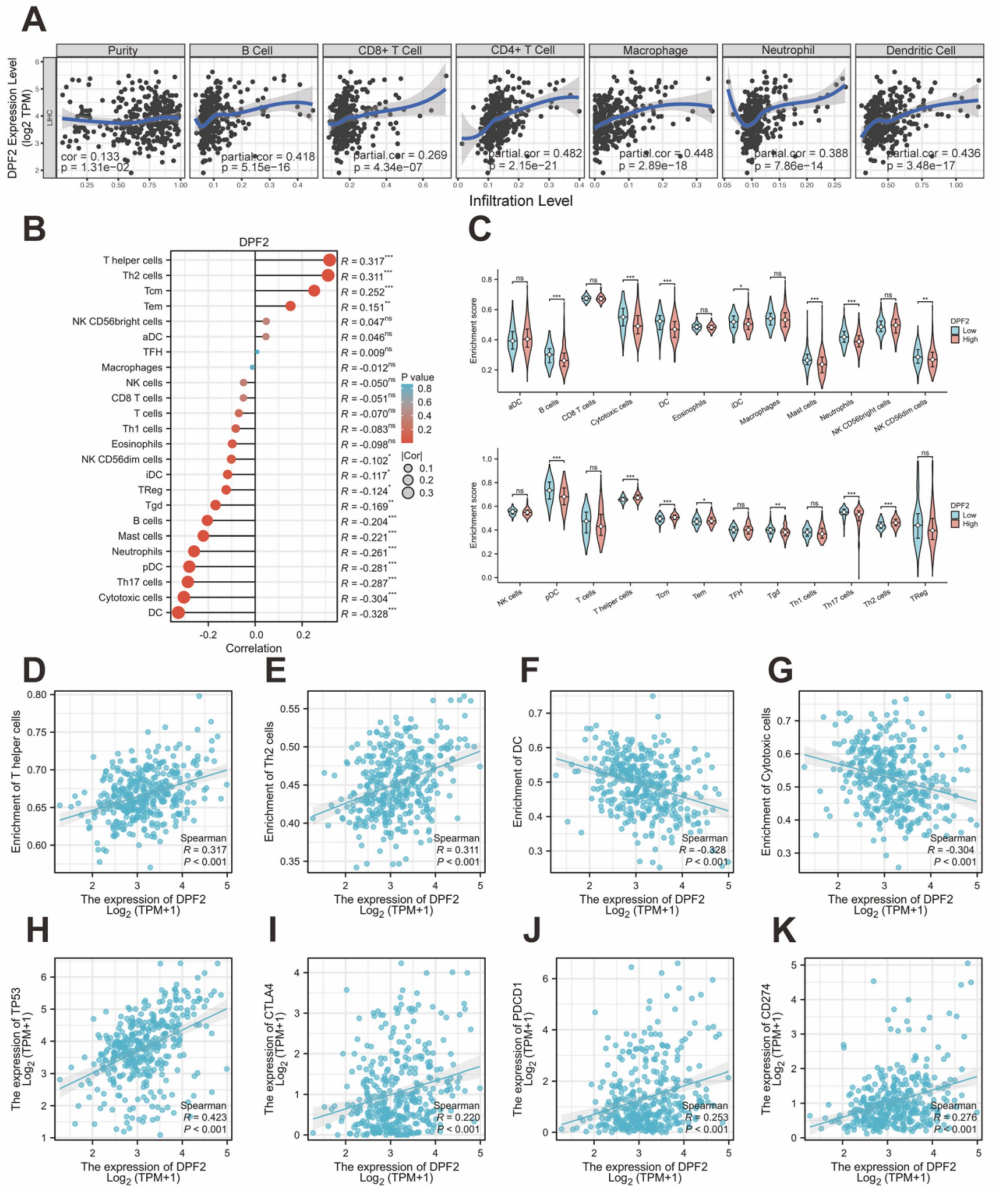
**Correlation of DPF2 expression with immune infiltration and immune checkpoints.** (A) Correlation of DPF2 in TIMER with tumor purity and immune cell infiltration status. (B) Bubble plot of the correlation between DPF2 and 24 immune cells. (C) Degree of immune infiltration of different immune cells in high and low DPF2 expression. Scatter plots of correlation between DPF2 expression levels and (D) T helper cells, (E) Th2 cells, (F) DCs and (G) cytotoxic cells. Scatter plots of correlation between DPF2 expression levels and (H) TP53, (I) CTLA-4, (J) PD-1 and (K) PD-L1. ns: p ≥ 0.05; *p < 0.05; **p < 0.01; ***p < 0.001.

It has been proved that immune cells play a significant role in cancer 39. Initially, we used TIMER to explore the relationship between DPF2 expression, tumor purity, and immune cell infiltration. The results revealed a significant positive correlation between DPF2 expression in HCC and tumor purity (r=0.133, p=1.81e-02), B-cell infiltration (r=0.418, p=5.15e-16), CD8+ T-cell infiltration (r=0.269, p=4.34e-07), CD4+ T-cell infiltration (r=0.482, p=2.15e-21), macrophage infiltration (r=0.448, p=2.89e-18), neutrophil infiltration (r=0.388, p=7.86e-14), and dendritic cell (DC) infiltration (r=0.436, p=3.48e-17) (Figure 9A).

Previous studies have emphasized the importance of intratumoral immune cells in human cancers 24. Our study examined the associations between DPF2 and 24 types of intratumoral immune cells using the ssGSEA algorithm. The analysis identified significant positive associations between DPF2 and T helper cells (r=0.317, p=4.27e-10), Th2 cells (r=0.311, p=1.01e-09), central memory T cells (Tcm) (r=0.252, p=8.90e-07), and effector memory T cells (Tem) (r=0.151, p=3.53e-03) (Figure 9B, D-E) (Figure S4). In contrast, DPF2 expression showed a significant negative correlation with dendritic cells (DC) (r=-0.328, p= 1.07e-10), cytotoxic cells (r=-0.304, p=2.32e-09), Th17 cells (r=-0.287, p=1.82e-08), plasmacytoid dendritic cells (pDC) (r=-0.281, p=3.29e-08), neutrophils (r =-0.261, p=3.51e-07), mast cells (r=-0.221, p=1.65e-05), B cells (r=-0.204, p=7.26e-05), gamma delta T cells (Tgd) (r=-0.169, p=1.05e-03), regulatory T cells (Treg) (r=-0.124, p=1.68e-02), immature dendritic cells (iDC) (r=-0.117, p=2.33e-02), and NK CD56dim cells (r=-0.102, p=4.97e-02) (Figure 9B, F-G) (Figure S4). We compared the enrichment scores of the 24 immune cells in groups with high and low DPF2 expression. The results indicated that the DPF2 high-expression group had higher enrichment scores in 4 types of immune cells, whereas the low DPF2 expression group exhibited higher scores in 11 types of immune cells (Figure 9C).

Genes with TP53 mutations have been linked to poor prognosis in HCC 40. Immune checkpoints play crucial roles in tumor immune evasion, and immune checkpoint inhibitors have become important therapeutic agents in liver cancer treatment 41. We further investigated the correlations between DPF2 and TP53, CTLA-4, PD-1 (PDCD-1), and PD-L1 (CD274) using the TCGA database. In HCC, DPF2 expression showed significant positive correlations with several key immune-related molecules. Specifically, we observed strong associations between DPF2 and TP53 (r=0.423, p<0.001), CTLA-4 (r=0.220, p<0.001), PD-1 (r=0.253, p<0.001), and PD-L1 (r=0.276, p<0.001) (Figure 9H-K).

### 3.9 Immunohistochemical verification of high DPF2 expression in HCC

**Figure 10:**
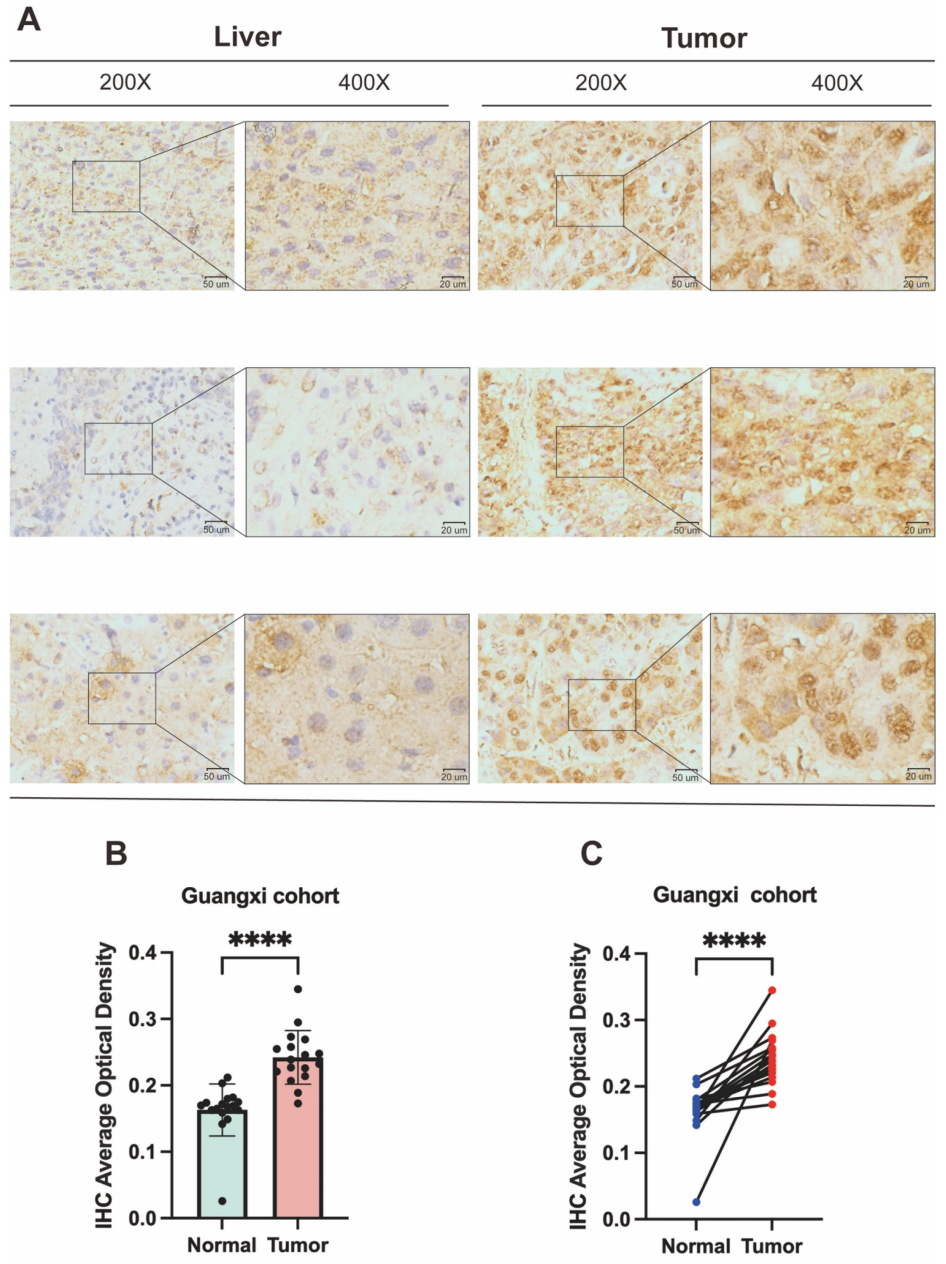
**Validation of DPF2 protein expression in Guangxi patients with hepatocellular carcinoma.** (A) Representative immunohistochemical (IHC) images of DPF2 expression in HCC and adjacent liver tissues. (B-C) Average optical density (AOD) of immunohistochemical staining of DPF2 in HCC and adjacent liver tissues. ****p < 0.0001.

To corroborate DPF2 expression in HCC patients, we performed immunohistochemical validation on 17 pairs of HCC and corresponding paracancerous tissues obtained from the First Affiliated Hospital of Guangxi Medical University. The findings indicated that DPF2 was primarily expressed in the nucleus and that its expression was higher in HCC tissues compared to paracancerous tissues (Figure 10A). Using a semi-quantitative evaluation method 42, 43, we calculated the average optical density (AOD) of DPF2 immunohistochemical (IHC). The results showed that the AOD of DPF2 in HCC tissues was significantly higher than in the surrounding non-cancerous tissues (p<0.0001) (Figures 10B-C).

## 4. Discussion

Hepatocellular carcinoma (HCC) is characterized by high morbidity, mortality, and heterogeneity, posing a significant threat to global health 44. According to the Cancer Statistics Report 2024, there has been a slight decrease in morbidity and mortality among male liver cancer patients. However, for female patients, both the morbidity and mortality rates of HCC continue to rise year after year 45. Typically, liver cancer patients are often diagnosed at an advanced stage, which significantly contributes to the disease's poor prognosis. This issue primarily stems from the lack of effective early diagnostic tools for liver cancer 46, 47. As a result, the search for effective biomarkers to improve early detection of HCC is urgently needed.

DPF2, a transcription factor, has been demonstrated in prior studies to activate the non-classical NF-κB pathway by binding to subunits of the SWI/SNF complex 48. NF-κB is persistently activated in various tumors 49, and inhibition of the SWI/SNF complex has been shown to suppress NF-κB activation, leading to tumor-suppressing effects 50. Consequently, DPF2 may be associated with certain cancers. Previous studies have confirmed that DPF2 is strongly linked with cancers such as glioblastoma 51 and acute myeloid leukemia 52. Our current research utilizes a pan-cancer analysis through the TCGA database, which revealed a significant up-regulation of DPF2 across 17 types of malignant tumors, primarily including conditions such as hepatocellular carcinoma, breast carcinoma, cholangiocarcinoma, pancreatic carcinoma, and melanoma among others. These findings align with the outcomes of an earlier study 15, suggesting that DPF2 may be a potential oncogenic gene for a broad range of cancers. In our analysis of DPF2 expression and prognosis in several hepatocellular carcinoma-related databases, DPF2 was found to be significantly overexpressed in both paired and unpaired group samples of HCC. This overexpression was strongly correlated with adverse clinicopathological features and poor prognosis in HCC. These results were further validated by immunohistochemistry experiments on the Guangxi hepatocellular carcinoma cohort, which confirmed the findings. Additionally, ROC analysis of several databases and the construction of a Nomogram model demonstrated that DPF2 has good diagnostic value, suggesting that DPF2 may serve as an effective diagnostic and prognostic biomarker for hepatocellular carcinoma.

Cancer development is closely linked to gene mutations and DNA methylation 53, 54. Various therapies for concurrent cancers have been shown to be strongly associated with ferroptosis 55, 56. To investigate the epigenetic mechanisms of DPF2's involvement in HCC development, this study analyzed the gene mutation and methylation of DPF2 and its interaction with ferroptosis-related genes. The analysis revealed that the frequency of DPF2 gene mutation in HCC was 1.3%, but the mutation of DPF2 was not significantly correlated with adverse outcomes in HCC. Methylation pattern analysis showed that DPF2 had higher methylation levels in HCC tissues than in adjacent normal liver tissues. Additionally, a correlation was observed between DPF2 hypermethylation and unfavorable prognosis in HCC, consistent with the prognostic outcomes of DPF2 in the transcriptome. Among the 25 genes associated with ferroptosis, DPF2 expression was positively associated with 20 genes and negatively correlated with 3 genes, suggesting that DPF2 may be involved in the ferroptosis-related process, thereby influencing the treatment and prognosis of hepatocellular carcinoma.

In this investigation, we further elaborated on the biological roles and associated pathways of DPF2. GO and KEGG analyses revealed that DPF2 predominantly functions in digestion, hormone activity, and neurotransmitter receptor activity. It plays a crucial role in signaling pathways, including neuroactive ligand-receptor interaction, protein digestion, and absorption. GSEA demonstrated a significant correlation between DPF2 and the cell cycle, Wnt signaling pathway, cancer pathways, DNA replication, immunoglobulin complex, immunoglobulin receptor binding, B-cell receptor signaling pathway, and antigen binding. Concurrently, we assessed genes closely linked to DPF2 and their biological roles through protein interaction network analysis, which revealed that DPF2 is chiefly associated with nucleosome disassembly, protein-DNA complex disassembly, SWI/SNF complex, and hepatocellular carcinoma. This suggests that DPF2 may contribute to the pathogenesis of hepatocellular carcinoma through immune-antigen binding, involvement in the cell cycle, Wnt signaling pathway, and cancer pathways, with the Wnt signaling pathway playing a pivotal role in the oncogenic activation of HCC 57. To validate these findings, we conducted a deeper investigation into the correlation between DPF2 and immune infiltrating cells as well as immune checkpoints. Our study revealed that DPF2 significantly enhanced the immune infiltration levels of T helper cells and Th2 cells, which are known to increase significantly in patients with hepatocellular carcinoma 58. Additionally, this study verified a significant correlation between DPF2 expression and TP53, CTLA-4, PD-1, and PD-L1. Consequently, DPF2 may facilitate the infiltration of immune cells, such as T helper cells and Th2 cells, and the expression of immune checkpoints, which mediate the immune evasion of hepatocellular carcinoma and foster its progression.

Concurrently, this investigation acknowledges certain limitations. Primarily, the expression and functional analysis of DPF2 was validated through an online database, without corroborating evidence from in vitro cellular experiments and in vivo animal studies. Therefore, it is imperative to enhance the relevant functional experiments to further authenticate the biological role of DPF2 in hepatocellular carcinoma in future research endeavors.

In conclusion, this investigation pioneered the analysis of the relationship between DPF2 and hepatocellular carcinoma. The study confirmed that DPF2 is notably overexpressed in HCC and shows a significant correlation with a poor prognosis. Furthermore, DPF2 may participate in immune infiltration, immune checkpoint evasion, and other related mechanisms in hepatocellular carcinoma. As a result, DPF2 could potentially be utilized as a biomarker for diagnosing and prognosticating hepatocellular carcinoma, and it might also be considered as a potential target for immunotherapeutic strategies.

## Supplementary Material

Supplementary figures and tables.

## Figures and Tables

**Table 1:** Relationship between DPF2 expression and clinicopathological features in the TCGA database.

Characteristics	Low expression of DPF2	High expression of DPF2	P value	X^2^
n	187	187		
Age, n (%)			0.070	3.283
<= 60	80 (21.4%)	97 (26%)		
> 60	107 (28.7%)	89 (23.9%)		
Missing	0	1		
Gender, n (%)			0.912	0.012
Female	60 (16%)	61 (16.3%)		
Male	127 (34%)	126 (33.7%)		
Missing	0	0		
Race, n (%)			0.193	3.292
Asian	71 (19.6%)	89 (24.6%)		
Black or African American	7 (1.9%)	10 (2.8%)		
White	99 (27.3%)	86 (23.8%)		
Missing	10	2		
BMI, n (%)			0.140	2.177
<= 25	82 (24.3%)	95 (28.2%)		
> 25	87 (25.8%)	73 (21.7%)		
Missing	18	19		
AFP(ng/ml), n (%)			**< 0.001**	**13.472**
<= 400	122 (43.6%)	93 (33.2%)		
> 400	20 (7.1%)	45 (16.1%)		
Missing	45	49		
Albumin(g/dl), n (%)			0.111	2.542
< 3.5	41 (13.7%)	28 (9.3%)		
>= 3.5	112 (37.3%)	119 (39.7%)		
Missing	34	40		
Prothrombin time, n (%)			0.440	0.597
<= 4	102 (34.3%)	106 (35.7%)		
> 4	48 (16.2%)	41 (13.8%)		
Missing	37	40		
Child-Pugh grade, n (%)			0.426	0.634
A	119 (49.4%)	100 (41.5%)		
B&C	10 (4.1%)	12 (5%)		
Missing	58	75		
Pathologic T stage, n (%)			0.391	3.006
T1	99 (26.7%)	84 (22.6%)		
T2	42 (11.3%)	53 (14.3%)		
T3	37 (10%)	43 (11.6%)		
T4	6 (1.6%)	7 (1.9%)		
Missing	3	0		
Pathologic N stage, n (%)			0.704	0.144
N0	120 (46.5%)	134 (51.9%)		
N1	1 (0.4%)	3 (1.2%)		
Missing	66	50		
Pathologic M stage, n (%)			0.614	0.253
M0	133 (48.9%)	135 (49.6%)		
M1	3 (1.1%)	1 (0.4%)		
Missing	51	51		
Pathologic stage, n (%)			0.152	5.283
Stage I	93 (26.6%)	80 (22.9%)		
Stage II	40 (11.4%)	47 (13.4%)		
Stage III	36 (10.3%)	49 (14%)		
Stage IV	4 (1.1%)	1 (0.3%)		
Missing	14	10		
Tumor status, n (%)			0.062	3.490
Tumor free	110 (31%)	92 (25.9%)		
With tumor	68 (19.2%)	85 (23.9%)		
Missing	9	10		
Residual tumor, n (%)			0.136	2.222
R0	168 (48.7%)	159 (46.1%)		
R1&R2	6 (1.7%)	12 (3.5%)		
Missing	13	16		
Histologic grade, n (%)			**< 0.001**	**18.236**
G1	40 (10.8%)	15 (4.1%)		
G2	91 (24.7%)	87 (23.6%)		
G3	49 (13.3%)	75 (20.3%)		
G4	4 (1.1%)	8 (2.2%)		
Missing	3	2		
Vascular invasion, n (%)			0.057	3.631
No	116 (36.5%)	92 (28.9%)		
Yes	49 (15.4%)	61 (19.2%)		
Missing	22	34		
Adjacent hepatic tissue inflammation, n (%)			0.610	0.990
None	64 (27%)	54 (22.8%)		
Mild	48 (20.3%)	53 (22.4%)		
Severe	9 (3.8%)	9 (3.8%)		
Missing	66	71		
OS event, n (%)			0.082	3.018
Alive	130 (34.8%)	114 (30.5%)		
Dead	57 (15.2%)	73 (19.5%)		
Missing	0	0		
DSS event, n (%)			0.345	0.892
No	148 (40.4%)	139 (38%)		
Yes	36 (9.8%)	43 (11.7%)		
Missing	3	5		
PFI event, n (%)			0.179	1.808
No	102 (27.3%)	89 (23.8%)		
Yes	85 (22.7%)	98 (26.2%)		
Missing	0	0		

Abbreviations: AFP, alpha-fetoprotein; OS, overall survival; DSS, disease-specific survival; PFI, progress free interval. Bold values are used to highlight statistical significance, and P <0.05. Missing cases: Some of TCGA clinical data are missing.

**Table 2:** Logistic regression analysis of DPF2 expression.

Characteristics	Total (N)	OR (95% CI)	P value
Age (> 60 vs. <= 60)	373	0.686 (0.456 - 1.032)	0.070
Gender (Male vs. Female)	374	0.976 (0.633 - 1.505)	0.912
Race (Black or African American&Asian vs. White)	362	1.461 (0.966 - 2.211)	0.073
BMI (> 25 vs. <= 25)	337	0.724 (0.472 - 1.112)	0.141
AFP(ng/ml) (> 400 vs. <= 400)	280	2.952 (1.633 - 5.334)	**< 0.001**
Child-Pugh grade (B&C vs. A)	241	1.428 (0.592 - 3.444)	0.428
Pathologic T stage (T3&T4 vs. T1&T2)	371	1.197 (0.748 - 1.916)	0.454
Pathologic N stage (N1 vs. N0)	258	2.687 (0.276 - 26.173)	0.395
Pathologic M stage (M1 vs. M0)	272	0.328 (0.034 - 3.197)	0.338
Pathologic stage (Stage III&Stage IV vs. Stage I&Stage II)	350	1.309 (0.809 - 2.119)	0.273
Histologic grade (G3&G4 vs. G1&G2)	369	2.011 (1.307 - 3.095)	**0.001**
Tumor status (With tumor vs. Tumor free)	355	1.495 (0.980 - 2.280)	0.062
Vascular invasion (Yes vs. No)	318	1.570 (0.986 - 2.499)	0.057

Bold values are used to highlight statistical significance, and P <0.05.
