# Supplementary material for: DPF2 overexpression correlates with immune infiltration and dismal prognosis in hepatocellular carcinoma
Source: J Cancer. 2024 Jul 2;15(14):4668–85. doi: 10.7150/jca.97437 (PMC11242344; doi:10.7150/jca.97437)
Supplement: Supplementary file 1 — Supplementary figures and tables. [file jcav15p4668s1.pdf]

Figure S1

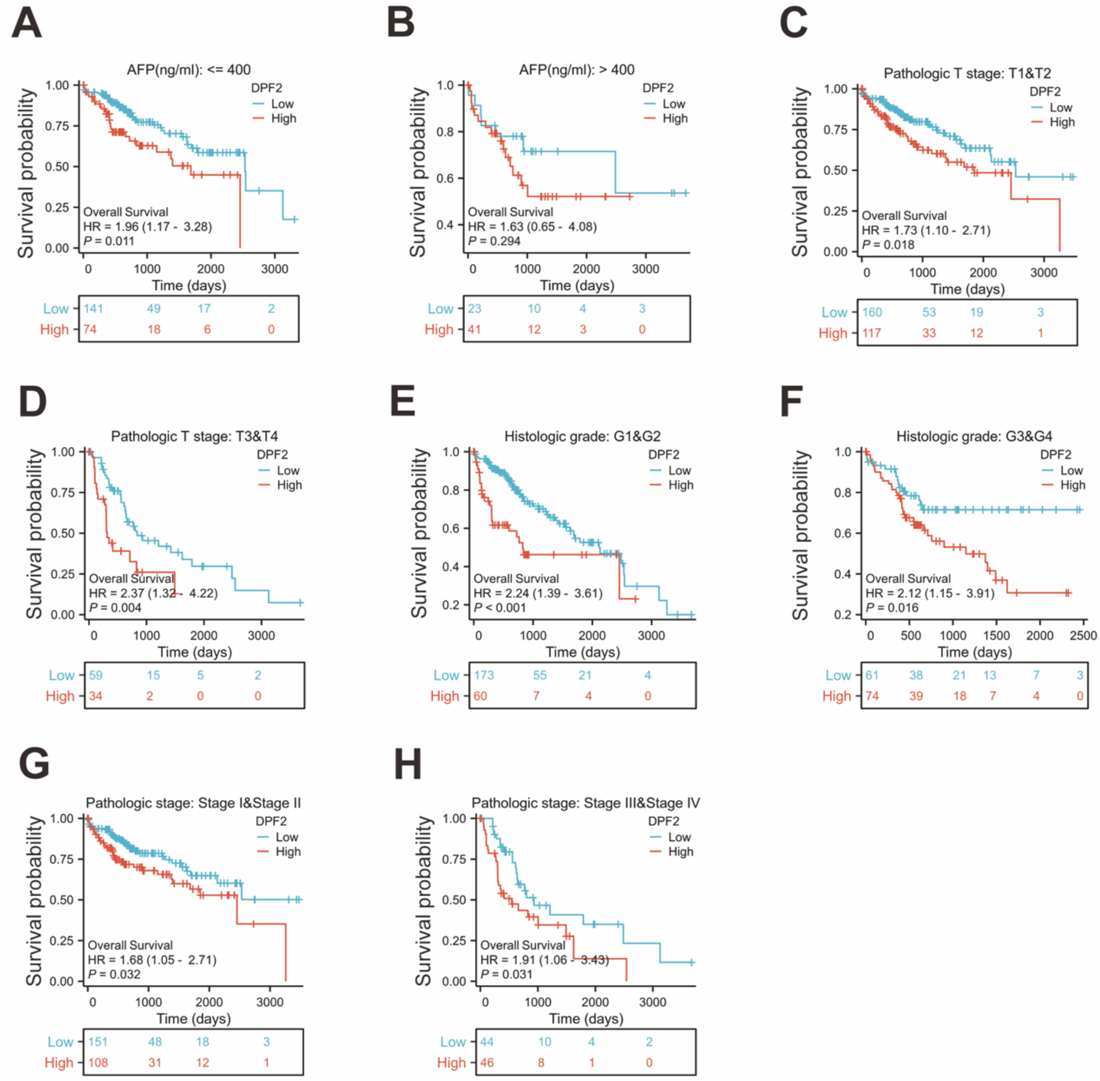

Figure S1. High expression of DPF2 was associated with poor prognosis. Kaplan–Meier survival curves of DPF2 in (A) AFP≤400, (B) AFP>400, (C) T1 and T2, (D) T3 and T4, (E) G1 and G2, (F) G3 and G4, (G) stage I and II and (H) stage III and IV subgroups. OS, Overall Survival. DSS, Disease Free Survival. PFI: Progression Free Interval. RFS, Recurrence Free Survival.

Figure S2

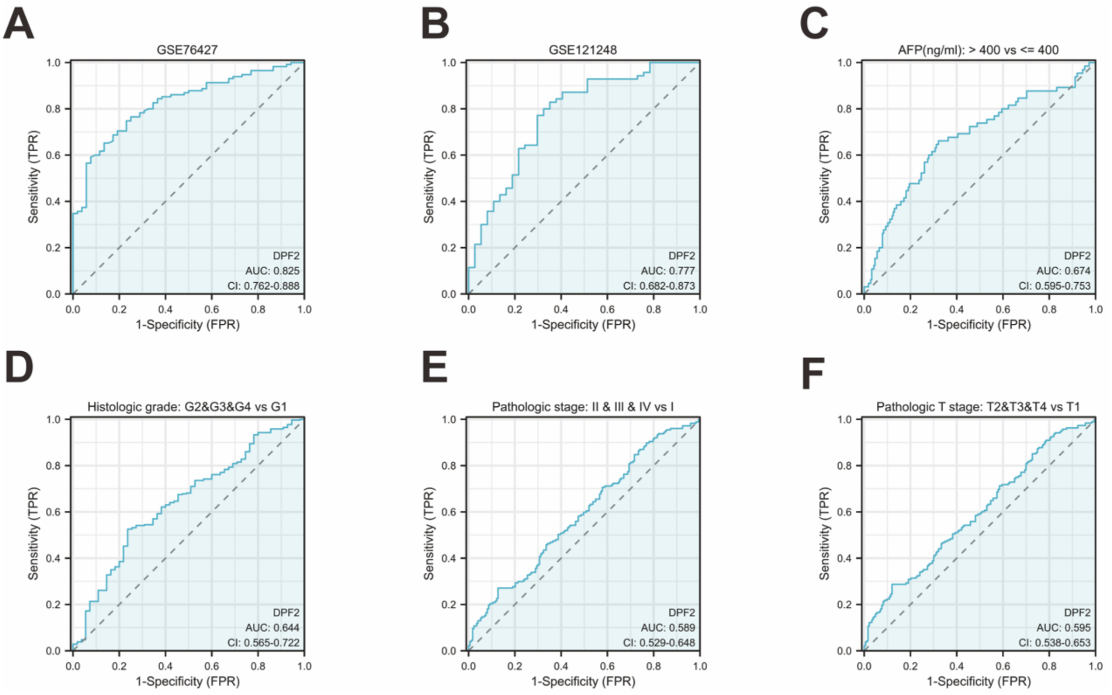

Figure S2. ROC validation of DPF2 in different databases and clinicopathological features. Diagnostic ROC curves in (A) GSE76427, (B) GSE121248, (C) AFP, (D) Histologic grade, (E) Pathologic stage, (F) T stage.

Figure S3

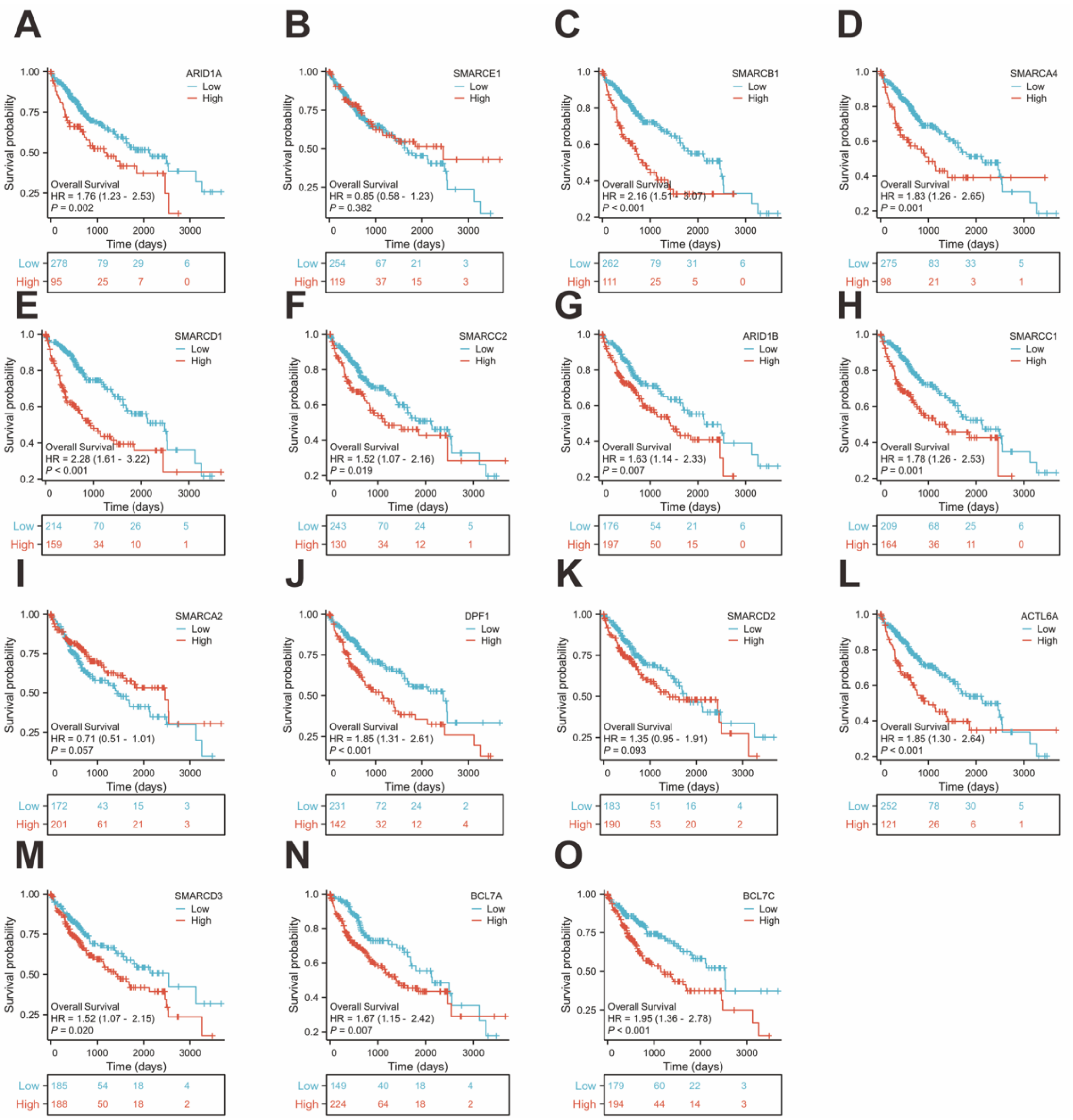

Figure S3. Prognosis of DPF2-related genes in hepatocellular carcinoma from the TCGA database. (A) ARID1A, (B) SMARCE1, (C) SMARCB1, (D) SMARCA4, (E) SMARCD1, (F) SMARCC2, (G) ARID1B, (H) SMARCC1, (I) SMARCA2, (J) DPF1, (K) SMARCD2, (L) ACTL6A, (M) SMARCD3, (N) BCL7A, (O) BCL7C. ns: $p \geq 0.05$; *$p < 0.05$; **$p < 0.01$; ***$p < 0.001$.

Figure S4

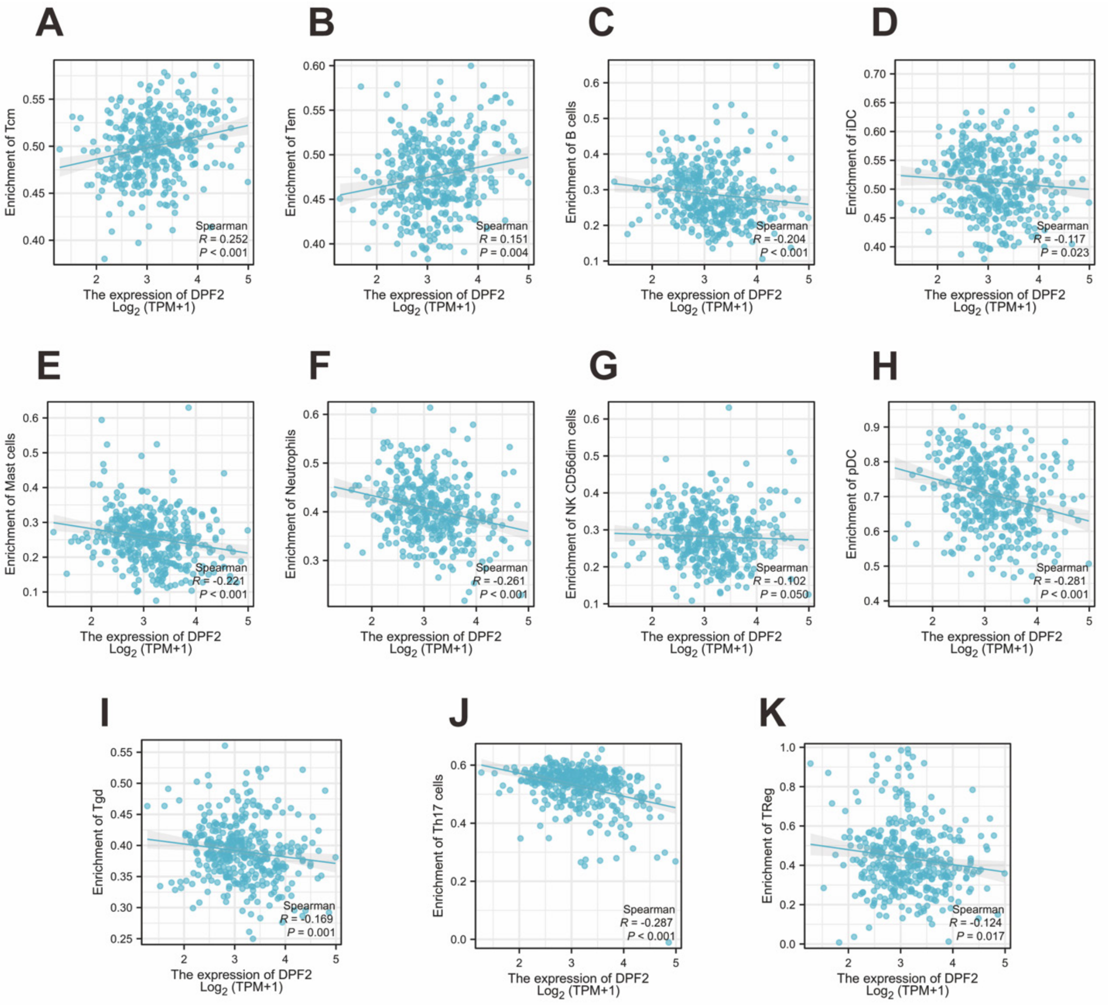

Figure S4. Supplementary Figure Correlation of DPF2 Expression with immune infiltration Cells. (A) Tcm, (B) Tem, (C) B cells, (D) iDC, (E) Mast cells, (F) Neutrophils, (G) NK CD56dim cells, (H) pDC, (I) Tgd, (J) Th17 cells, (K) Treg. ns: p ≥ 0.05; *p < 0.05; **p < 0.01; ***p < 0.001.

Table S1. Relationship between DPF2 expression and clinicopathological features in the GSE14520_3921 database.

Characteristics	Low expression of DPF2	High expression of DPF2	P value
n	110	111	
Age, n (%)			0.280
≤60	87 (39.4%)	94 (42.5%)	
>60	23 (10.4%)	17 (7.7%)	
Missing	0	0	
Gender, n (%)			0.675
M	94 (42.5%)	97 (43.9%)	
F	16 (7.2%)	14 (6.3%)	
Missing	0	0	
ALT(>/<=50U/L), n (%)			0.240
low	69 (31.2%)	61 (27.6%)	
high	41 (18.6%)	50 (22.6%)	
Missing	0	0	
AFP (>/<=300ng/ml), n (%)			**0.020**
low	67 (30.7%)	51 (23.4%)	

Characteristics	Low expression of DPF2	High expression of DPF2	P value
high	41 (18.8%)	59 (27.1%)	
Missing	2	1	
Main Tumor Size (>/<=5 cm), n (%)			0.702
small	68 (30.9%)	72 (32.7%)	
large	41 (18.6%)	39 (17.7%)	
Missing	1	0	
Multinodular, n (%)			0.640
N	89 (40.3%)	87 (39.4%)	
Y	21 (9.5%)	24 (10.9%)	
Missing	0	0	
Cirrhosis, n (%)			0.637
N	8 (3.6%)	10 (4.5%)	
Y	102 (46.2%)	101 (45.7%)	
Missing	0	0	
TNM staging, n (%)			0.467
I	51 (23.3%)	42 (19.2%)	

Characteristics	Low expression of DPF2	High expression of DPF2	P value
II	35 (16%)	42 (19.2%)	
III	24 (11%)	25 (11.4%)	
Missing	0	2	
BCLC staging, n (%)			0.973
0	11 (5%)	9 (4.1%)	
A	74 (33.8%)	74 (33.8%)	
B	11 (5%)	11 (5%)	
C	14 (6.4%)	15 (6.8%)	
Missing	0	2	
Survival status, n (%)			0.234
Alive	72 (32.6%)	64 (29%)	
Dead	38 (17.2%)	47 (21.3%)	
Missing	0	0	
Recurr status, n (%)			0.253
No	54 (24.4%)	46 (20.8%)	
Yes	56 (25.3%)	65 (29.4%)	

Characteristics	Low expression of DPF2	High expression of DPF2	P value
Missing	0	0	

Abbreviations: AFP, alpha-fetoprotein. Bold values are used to highlight statistical significance, and P <0.05. Missing cases：Some of GSE14520_3921 clinical data are missing.

Table S2. Relationship between DPF2 expression and clinicopathological features in the GSE14520_571 database.

Characteristics	Low expression of DPF2	High expression of DPF2	P value
n	10	11	
Age, n (%)			0.635
>60	2 (9.5%)	4 (19%)	
≤60	8 (38.1%)	7 (33.3%)	
Missing	0	0	
ALT(>/<=50U/L), n (%)			1.000
low	6 (28.6%)	6 (28.6%)	
high	4 (19%)	5 (23.8%)	
Missing	0	0	
Main Tumor Size (>/<=5 cm), n (%)			1.000
small	6 (28.6%)	7 (33.3%)	
large	4 (19%)	4 (19%)	

Characteristics	Low expression of DPF2	High expression of DPF2	P value
Missing	0	0	
Multinodular, n (%)			1.000
N	7 (33.3%)	7 (33.3%)	
Y	3 (14.3%)	4 (19%)	
Missing	0	0	
TNM staging, n (%)			0.600
I	1 (16.7%)	2 (33.3%)	
II	1 (16.7%)	0 (0%)	
III	0 (0%)	2 (33.3%)	
Missing	8	7	
BCLC staging, n (%)			0.467
A	2 (33.3%)	2 (33.3%)	
B	0 (0%)	2 (33.3%)	
Missing	8	7	
AFP (>/<=300ng/ml), n (%)			0.656
high	4 (20%)	6 (30%)	

Characteristics	Low expression of DPF2	High expression of DPF2	P value
low	6 (30%)	4 (20%)	
Missing	0	1	
Survival status, n (%)			0.395
Alive	6 (28.6%)	4 (19%)	
Dead	4 (19%)	7 (33.3%)	
Missing	0	0	
Recurr status, n (%)			0.361
No	4 (19%)	2 (9.5%)	
Yes	6 (28.6%)	9 (42.9%)	
Missing	0	0	

Abbreviations: AFP, alpha-fetoprotein. Bold values are used to highlight statistical significance, and $P < 0.05$. Missing cases：Some of **GSE14520_571** clinical data are missing.
